# Impact of genotyping errors on the type I error rate and the power of haplotype-based association methods

**DOI:** 10.1186/1471-2156-10-3

**Published:** 2009-01-29

**Authors:** Vivien Marquard, Lars Beckmann, Iris M Heid, Claudia Lamina, Jenny Chang-Claude

**Affiliations:** 1 Department of Cancer Epidemiology, German Cancer Research Center, Heidelberg, Germany; 2 Helmholtz Zentrum München, German Research Center for Environmental Health, Institute of Epidemiology, Neuherberg, Germany; 3 Ludwig-Maximilians-University Munich, Chair of Epidemiology, Munich, Germany; 4 Division of Genetic Epidemiology, Department of Medical Genetics, Molecular and Clinical Pharmacology, Innsbruck Medical University, Innsbruck, Austria; 5 Im Neuenheimer Feld 280, D-69120 Heidelberg, Germany

## Abstract

**Background:**

We investigated the influence of genotyping errors on the type I error rate and empirical power of two haplotype based association methods applied to candidate regions. We compared the performance of the Mantel Statistic Using Haplotype Sharing and the haplotype frequency based score test with that of the Armitage trend test.

Our study is based on 1000 replication of simulated case-control data settings with 500 cases and 500 controls, respectively. One of the examined markers was set to be the disease locus with a simulated odds ratio of 3. Differential and non-differential genotyping errors were introduced following a misclassification model with varying mean error rates per locus in the range of 0.2% to 15.6%.

**Results:**

We found that the type I error rate of all three test statistics hold the nominal significance level in the presence of nondifferential genotyping errors and low error rates. For high and differential error rates, the type I error rate of all three test statistics was inflated, even when genetic markers not in Hardy-Weinberg Equilibrium were removed. The empirical power of all three association test statistics remained high at around 89% to 94% when genotyping error rates were low, but decreased to 48% to 80% for high and nondifferential genotyping error rates.

**Conclusion:**

Currently realistic genotyping error rates for candidate gene analysis (mean error rate per locus of 0.2%) pose no significant problem for the type I error rate as well as the power of all three investigated test statistics.

## Background

The influence of measurement errors in explanatory variables on the properties of a test statistic, like its type I error rate or power, has always been important to investigate. For example, Bross [1] discovered that in a case-control study with nondifferential errors independent of the disease status, the type I error rate of the Chi-Squared test is not increased, whereas the power to detect an association is reduced.

In genetic association studies, where a large amount of genotype data is produced, measurement errors in the data are almost inevitable. Genotyping errors are reported to occur with different frequencies and for different reasons [2-4]. For candidate region association studies they are mostly caused for example by contamination of the DNA extract, low quality reagents or by human artefacts, and occur with a frequency between 0.1% and 15% [3,4]. They are known to influence important issues, like the selection of tagging SNPs [5,6] or haplotype frequency estimation [7-9], and the properties of test statistics, e.g. [10,11]. For family based studies, it has been shown that the false-positive rate (type I error rate) of the Transmission/Disequilibrium Test (TDT) is dramatically inflated due to undetectable genotyping errors causing an overtransmission of common alleles [12]. Therefore, test statistics that account for genotyping errors have been developed, e.g. TDTae [13,14]. Additionally, methods to detect and deal with genotyping errors [3,15,16] have been of great interest, for example the use of double-sampling procedures [17,18]. Furthermore, it is still a matter of controversy as to whether deviation from Hardy-Weinberg-Equilibrium (HWE) should be used to identify genotyping errors [19-21].

So far, few studies have investigated the impact of genotyping errors on haplotype-based association methods, while there are reports on their impact on haplotype frequency estimation. It was previously shown that the type I error rate of a haplotype-based TDT (HS-TDT) is inflated in the presence of genotyping errors [22], but that the test statistic can be robustified [23]. Moskvina et al. [24] discovered in a theoretical approach that "genotyping errors tend to make the genotype distribution more similar to the stable distribution", which in genetic case-control association studies generally leads to a loss in power for nondifferential errors and to an increased type I error rate in the presence of differential errors. The type I error rate of a likelihood ratio test of independence of haplotype frequency and affection status, based on two marker haplotypes, was examined in a simulation study and found to be inflated given differential genotyping errors even with error rates lower than 1% for markers with small minor allele frequencies (MAF) and markers in strong LD [25].

Our aim is to explore the impact of genotyping errors on the type I error rate and the power of two haplotype-based association test statistics for candidate regions. The commonly used haplotype-based score test (haplo.score, [26]) relies on estimates of haplotype frequencies. Thus, since the influence of genotyping errors on haplotype frequency estimation is known, they might also have a large impact on the performance of haplo.score. The other haplotype-based test statistic is the Mantel Statistic Using Haplotype Sharing [27], which is of particular interest, since haplotype-sharing based association methods have not been investigated in the presence of genotyping errors. Haplotype-sharing is a nonparametric approach that does not rely on estimates of haplotype frequencies and assumptions on the underlying disease models, and may thus be more robust against errors concerning the haplotype distribution. Both investigated test statistics use haplotype information, but the Mantel Statistic Using Haplotype Sharing is actually a pointwise test. Therefore, we additionally investigated the single-point Armitage trend test as a comparison.

We simulated case-control scenarios with differential and nondifferential genotyping errors, incorporated by following the unrestricted misclassification model, proposed by Heid et al. [28]. Heid et al. estimated the occurrence of genotyping errors by assessing genotypes in duplicates and fitting several misclassification models given as the 3 × 3 misclassification matrix. We used their observed mean error rate per locus of around 0.2% in a simulation scenario to analyze the influence of a realistic amount of genotyping errors, and additionally, investigated higher error rates of 8 and 15.6% that are in the range of rates already used in previous simulation studies [3,19,29]. The simulated haplotype data was based on haplotypes across 15 SNPs as described in Heid et al. [30] to provide realistic haplotype scenarios. We estimated the type I error rate and empirical power for the Mantel Statistic Using Haplotype Sharing [27] and the haplotype-based score test (based on haplotype frequencies) [26]) and compared them with those of the Armitage trend test for all examined scenarios.

## Methods

### Data

The simulated data sets were based on a real haplotype distribution including 18 different haplotypes with a frequency of > 1%, describing a region in the *APM1 *gene, the adiponectin encoding gene [30]. We standardized the given estimated haplotype frequencies to achieve an overall frequency of 1 (see Table 1). Each haplotype consists of 15 carefully selected tagging SNPs. We simulated genotype data for different scenarios, containing either no, differential or nondifferential genotype errors, respectively. For each scenario, we generated 1000 replications with each 500 cases and 500 controls. Two haplotypes were drawn randomly to form an individual. For the analysis of type I error rate, without loss of generality the first 500 randomly drawn haplotype pairs were chosen to be cases and the last 500 haplotype pairs to be controls. For the analysis of empirical power, case-control status was assigned based on a logistic regression model with a recessive mode of inheritance. Here, we assumed a baseline odds ratio for the disease to be 1.017 and an odds ratio for carriers with two copies of the disease allele to be 3. Via the logistic regression model a probability to develop the disease based on their genetic components can be determined for each individual. According to these probabilities an individual was stated to be a case or a control. Haplotype pairs were drawn until the sample size of 500 cases and 500 controls was obtained. Marker 13 with a minor allele frequency of 0.028 (see Table 1) was chosen to be the putative disease locus.

**Table 1:** Haplotype distribution

Haplotypes															Frequencies
0	0	0	0	0	0	0	0	0	0	0	0	0	0	0	0.026
0	0	0	1	1	0	0	0	0	0	0	0	0	0	0	0.021
0	0	0	1	1	1	0	0	0	0	0	0	0	1	0	0.012
0	0	0	1	1	0	0	0	0	0	0	0	0	1	0	0.035
0	0	0	0	0	0	0	0	0	0	0	0	0	1	0	0.074
0	0	0	1	1	0	0	0	1	0	1	1	0	0	0	0.044
0	0	0	1	1	0	0	0	1	0	0	0	0	1	0	0.139
0	0	0	1	0	1	1	0	1	0	0	0	0	1	0	0.049
0	1	0	0	0	1	1	0	1	0	0	0	0	1	0	0.108
0	0	0	0	1	0	0	0	0	0	1	1	0	0	0	0.011
0	0	0	0	0	0	0	1	1	0	1	1	0	0	0	0.061
0	0	0	0	0	0	0	0	1	0	1	1	0	0	0	0.112
0	0	0	0	0	0	0	0	0	1	0	1	0	0	1	0.074
0	0	0	1	1	0	0	0	1	0	0	0	1	1	0	0.028
0	0	0	0	0	0	0	0	1	0	0	0	0	1	0	0.105
0	0	1	0	0	0	0	0	1	0	1	1	0	0	0	0.021
1	0	1	0	0	0	0	0	1	0	1	1	0	0	0	0.060
1	0	1	0	0	0	0	0	0	1	0	1	0	0	1	0.018

0.078	0.108	0.099	0.329	0.29	0.338	0.157	0.061	0.727	0.074	0.309	0.401	0.028	0.55	0.092	MAF*

* MAF – Minor Allele Frequency

Haplotype distribution used in simulation studies. The distribution is based on Heid et al. [30].

### Genotyping error

Genotype misclassification was incorporated independently at every marker for a haplotype pair following the unrestricted misclassification model described by Heid et al. [28]. A SNP genotype misclassification model is described by a 3 × 3 misclassification matrix with each cell containing the probability to assess a true genotype of 0, 1, 2 (coding the number of minor alleles of a SNP) as an observed genotype of 0, 1, 2. The mean error rate per locus is then calculated as the sum of frequencies of all discordant genotypes [3].

Table 2 shows the relative frequencies of possible genotyping errors corresponding to a mean error rate per locus of 0.2%, 8% and 15.6%, respectively. For example, a mean error rate of 0.2% is achieved when a true homozygote major allele genotype (0) is correctly classified with a relative frequency of 0.99951, whereas it is misclassified as a heterozygote (1) or a homozygous minor allele genotype (2) with a relative frequency of 0.00039 or 0.0001, respectively.

**Table 2:** Misclassification matrix

		Observed genotype			Mean error rate per locus (%)
		0	1	2	
	
	0	0.99951	0.00039	0.00010	
	1	0.00243	0.99602	0.00155	0.2
	2	0.00138	0.00023	0.99839	
	
True Genotype	0	0.975	0.018	0.007	
	1	0.02	0.97	0.01	8
	2	0.01	0.018	0.972	
	
	0	0.95	0.039	0.011	
	1	0.035	0.945	0.02	15.6
	2	0.015	0.036	0.949	

Misclassification matrix for the unrestricted error model with mean error rate per locus of 0.2%, 8% and 15.6%. The homozygous major allele genotype is coded as 0, the heterozygote as 1 and the homozygous minor allele genotype as 2. For each true genotype (0, 1, 2), the relative frequencies to observe the corresponding true or false genotypes are presented. Misclassification probabilities for an error rate per locus of 0.2% are based on Heid et al. [28].

In previous studies, different genotype mean error rates per locus between 0.1% and 15% have been reported and used in simulation studies [3,19,29]. Therefore, we investigated the influence of genotyping errors with the observed rate of 0.2% by Heid et al. [28], but also high error rates of 8% and 15.6%. The influence of nondifferential errors was investigated by incorporating genotype errors in cases and controls following the same error model with the same misclassification probabilities. Differential genotype errors might occur, e.g. when cases and controls were genotyped in different laboratories or when data from different sites or different populations were combined. These differential errors were simulated by introducing errors (1) in cases, but not in controls, or (2) in the first seven markers in cases and in the last seven markers in controls.

### Association statistics

We compared the type I error rate and the empirical power of three different statistics testing association in candidate regions or genes.

#### Mantel Statistic Using Haplotype Sharing

We applied the pointwise Mantel Statistic Using Haplotype Sharing [27] which uses the information of neighbouring markers. It correlates genetic and phenotypic similarity across all pairs of haplotypes, where the genetic similarity is measured as the shared length between haplotypes and the phenotypic similarity is the mean-corrected cross product based on the coded phenotypes (the disease status). Significance was assessed by Monte Carlo permutation of the disease status, while the haplotype pair of an individual was kept together.

Haplotypes used for the Mantel Statistic Using Haplotype Sharing were estimated from the genotypes via the EM algorithm, implemented in R [31]. For comparison we additionally estimated haplotypes via fastPHASE [32].

#### Armitage Trend Test

The second pointwise test we applied was the Armitage trend test. The Armitage trend test is a 2 × 3 (1 df) Chi-squared test for independence and was calculated via logistic regression with the count of the minor alleles (0, 1, 2) as the independent variable.

#### Haplo.score

The third statistic we used was a haplotype-based score test (haplo.score, [26,31]). The test describes haplotype association in a generalized linear model framework. It is carried out under the global null hypothesis of no haplotype association and relies on the probability distribution of haplotype pairs conditional on the observed individual genotype.

### Test for deviation from Hardy-Weinberg Equilibrium

We used the standard asymptotic Chi-squared test with 1 degree of freedom to test for deviations from Hardy-Weinberg-Equilibrium at a nominal significance level of 0.05 ([33], p.64f). This test was applied to each marker for each replication on controls only. As a data quality check on genotyping errors, all markers not in Hardy-Weinberg-Equilibrium (HWE) were then excluded from the analysis for the corresponding replication.

### Type I error rate and power

For each replication, we determined the pointwise p-value for the Armitage trend test and the Mantel Statistic Using Haplotype Sharing as well as the global p-value for the haplo.score test. Significance was then defined with a p-value less than the significance level of α = 0.05. The number of significant replications was counted and divided by the number of total replications. When no disease locus was simulated, this number reflects the type I error rate. If there is a disease locus, it represents the empirical power at the disease locus.

## Results

### Quality of haplotype estimation

#### Comparison of estimated haplotypes based on genotypes with or without errors

We counted the number of correctly estimated SNPs per haplotype as a measure for quality of haplotype estimation. When no genotyping errors or errors with a low error rate of 0.2% are incorporated, we observed for both phasing algorithms that nearly all 1000 haplotype pairs (for each replication) are estimated correctly. With increasing genotype error rate, the number of accurately estimated haplotypes decreases. For an error rate of 8%, on average 800 haplotype pairs are correctly estimated, whereas with an error rate of 15.6%, only 600 are. This effect is independent of differential and non-differential error scenarios. However, at least 10 of 15 SNPs are estimated correctly for all scenarios, i.e. the estimated haplotypes did not differ from the true haplotypes (without genotype errors) at more than five SNPs

#### Estimated haplotype frequencies and number of estimated haplotypes

**Figure 1:**
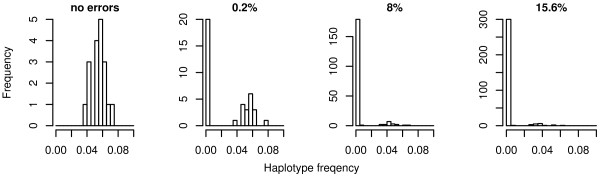
**Estimated haplotype distributions**. Number of estimated haplotypes and their distribution according to haplotype frequencies for different error rates. The number of rare haplotypes is increased with increasing mean error rate per locus.

When genotype errors are not incorporated, all data sets consist of 18 different haplotypes with frequencies between 0.011 and 0.139. For both haplo.em and fastPHASE, the number of different haplotypes increases drastically with increasing genotyping error rates, as shown for haplo.em in Figure 1. With a mean error rate per locus of 0.2%, 38 different haplotypes can be observed, of which 20 have a frequency of less than 1%. With higher error rates of 8% and 15.6%, the number of different haplotypes becomes 198 and 319, respectively, most of which have a frequency of less than 1%. Again this is observed for differential and non-differential error scenarios. The large number of rare haplotypes seems to be the reason for the influence of genotyping errors on haplotype frequency based association test statistics and has also an influence on the computational time to determine haplotypes via the EM-algorithm or fastPHASE.

### Type I error rate is inflated only for differential genotyping error rates

**Figure 2:**
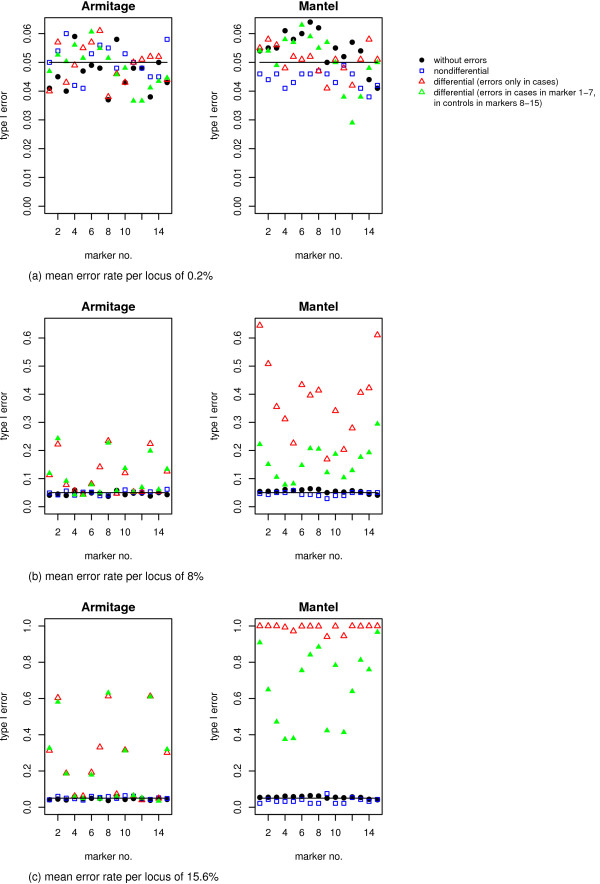
**Results on Type I error rate**. Type I error rate of the Armitage trend test and the Mantel Statistic Using Haplotype Sharing with no (dots), differential (triangulars) and nondifferential (squares) genotype errors incorporated following the unrestricted misclassification model for different error rates per locus. With a small error rate of a) 0.2% both pointwise tests hold the nominal significance level of 0.05 (horizontal line), but with a higher error rate of b) 8% or c) 15.6% the type I error rate is dramatically increased for differential genotyping errors.

Figure 2 shows the calculated type I error rate for the pointwise tests, the Mantel Statistic Using Haplotype Sharing and the Armitage trend test. When genotyping errors are incorporated with a mean error rate per locus of 0.2%, both association test statistics achieve the nominal significance level of 5% at every marker, independent of the presence of genotyping errors (see Figure 2a). When genotyping errors are incorporated with a higher error rate of 8% (see Figure 2b) or 15.6% (see Figure 2c), the type I error rate of both the Armitage trend test and the Mantel Statistic Using Haplotype Sharing is increased in the presence of differential genotyping errors. For the Armitage trend test it increases to 0.25 or 0.6, respectively and the type I error rate of the Mantel Statistic Using Haplotype Sharing increases to 0.65 and nearly 0.99, respectively. The global type I error rate of the haplotype specific score test (haplo.score) is also only inflated in the presence of differential genotyping errors appearing with the higher rates of 8% or 15.6%, see Table 3.

**Table 3:** Results on Type I error rate of haplo.score

Mean error rate per locus (%)	Nondifferential	Differential (errors only in cases)	Differential (cases: first 7 markers, controls: last 7 markers)
Without errors	0.047		
0.2	0.047	0.384	0.057
8	0.049	1	0.333
15.6	0.037	1	0.973

Type I error rate of the haplotype based score test (haplo.score) for the unrestricted model with different mean error rates per locus.

**Figure 3:**
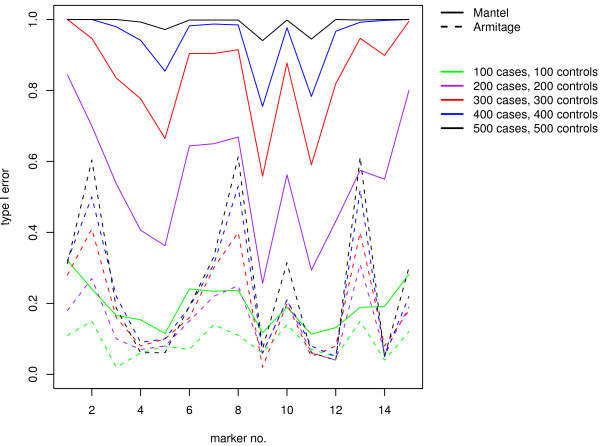
**Results on Type I error rate in dependence on sample size**. Type I error rate of the Armitage trend test (dotted lines) and the Mantel Statistic Using Haplotype Sharing (dashed lines) in the presence of differential genotyping errors (5% in cases, no errors in controls) for different sample sizes (100, 200, 300, 400, 500 case-control pairs).

Further investigations show that this dramatic increase in the type I error rate for differential genotyping errors is strongly dependent upon sample size. The inflation of the type I error rate of the Mantel Statistic Using Haplotype Sharing as well as of the Armitage trend test increases with sample size as it can be seen in Figure 3 for a mean error rate per locus of 15.6%, when genotyping errors were incorporated in cases only. The incorporation of differential genotyping errors in cases in the first seven markers and in controls in the last seven markers yields the same results (data not shown). The haplotype-based score test, haplo.score, rejects always the null hypothesis for all investigated sample sizes when genotyping errors are present in cases only. When differential genotyping errors are in cases in the first seven markers and in controls in the last seven markers, the type I error rate of the haplo.score test is increased with increasing sample sizes. With a sample size of 100 the type I error rate is 0.07, but for a sample size of 300 it is 0.34 and for a sample size of 500 0.72. Hence, an increase of the type I error rate can be seen, but is not as high as for differential errors in cases only. When genotype errors are present with a mean error rate per locus of 0.2%, the type I error rate of all three association test statistics remain at the nominal level of 0.05, independent of sample size.

In summary, the type I error rate is only increased for higher genotyping error rates with differential genotyping errors, but its magnitude depends on the sample size.

### Power is reduced in the presence of high, non-differential genotype error

**Figure 4:**
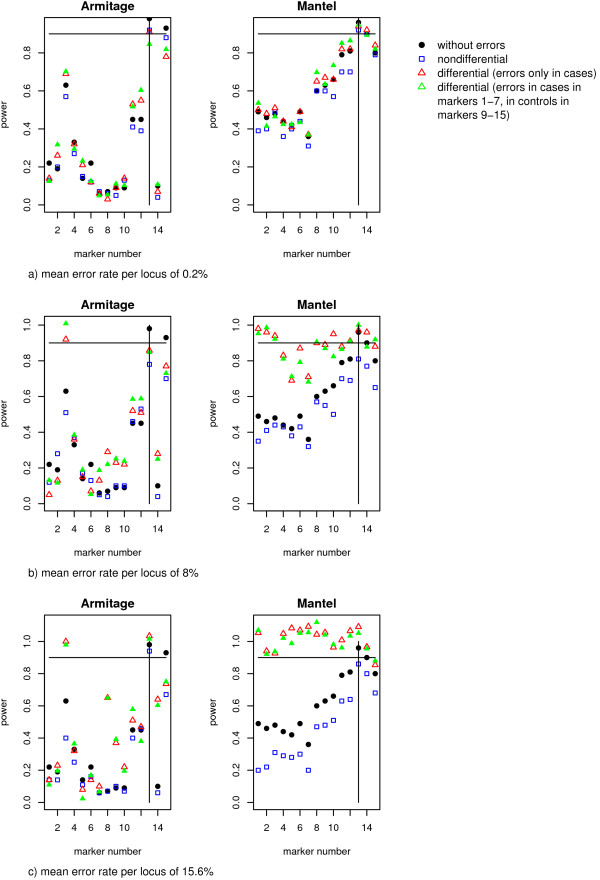
**Results on empirical power**. Empirical power of the Armitage trend test and the Mantel statistic Using Haplotype Sharing for no (dots), differential (triangulars) and nondifferential (squares) genotype errors incorporated following the unrestricted misclassification model for different error rates per locus. With a small error rate of a) 0.2% both pointwise tests achieve a power of around 90–100%. With higher error rates of b) 8% or c) 15.6% the power is reduced for nondifferential genotyping errors.

For small genotyping error rates of 0.2%, the empirical power of all three association test statistics was unaffected by nondifferential or differential genotyping errors. For haplo.score, power remains at 89–92%. The Armitage trend test and the Mantel Statistic Using Haplotype Sharing have a power of 92% to 94% to detect the disease locus (see Figure 4a). The influence of genotype errors with higher mean error rates per locus of 8% (Figure 4b) or 15.6% (Figure 4c) on the two pointwise tests are shown in Figure 4b and 4c, respectively. Here, the empirical power at the disease locus (marker 13) is decreased for the Armitage trend test and the Mantel Statistic Using Haplotype Sharing for non-differential genotyping errors to achieve only a level of around 80%. This decrease in power for nondifferential genotype errors is also observed for haplo.score, which achieves, for example, only a level of 48% in the presence of genotyping errors with an error rate of 15.6%. Furthermore, Figure 4b and 4c show that the power of the Mantel Statistic Using Haplotype Sharing is increased for differential genotyping errors, which might be due to the inflated type I error rates.

In summary, with low genotyping error rates, the empirical power of all three association test statistics remains high at around 89% to 94%. But power is decreased by high, nondifferential genotype errors.

### Test for deviation from Hardy-Weinberg Equilibrium

With a mean error rate per locus of 8% and a sample size of 1000, genotype errors are expected to occur on average in 80 individuals per marker. We observed that genotype errors occurred in each of the markers for all replications. Table 4 presents the number of replications where the marker was tested to deviate significantly from the assumption of Hardy-Weinberg Equilibrium for the scenario of non-differential genotype errors with a mean error rate per locus of 8%. Thus, the proportion of genotype errors detected by testing for deviation from HWE can be quite low, especially for markers with common alleles. This is also observed for all other error rates, for differential as well as for non-differential genotype errors (data not shown).

**Table 4:** Amount of genotype errors

SNP	1	2	3	4	5	6	7	8	9	10	11	12	13	14	15
Number of replications with at least 1 genotype error	1000	1000	1000	1000	1000	1000	1000	1000	1000	1000	1000	1000	1000	1000	1000
Number of replications with deviation from HWE	804	534	830	43	58	122	247	602	64	424	69	108	874	80	437

For each SNP the number of replications where a genotype error occurs in at least one person compared with the number of replications with a significant deviation from Hardy-Weinberg Equilibrium (p-value < 0.05). These numbers have been calculated for the scenario of nondifferential genotype errors with a mean error rate per locus of 8%.

**Figure 5:**
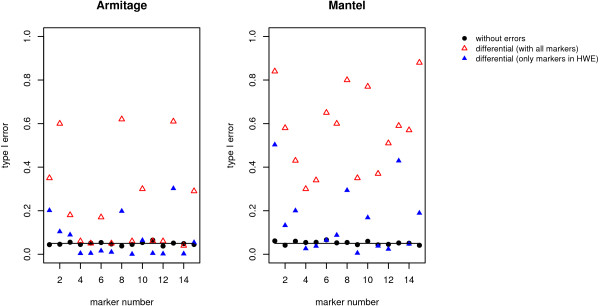
**Results on Type I error rate regarding deviations from HWE**. Type I error rate of the Armitage trend test and the Mantel Statistic Using Haplotype Sharing with no (dots) and differential (triangulars) genotyping errors (mean error rate per locus 15.6%). The type I error rate is only slightly decreased, when markers not in HWE are excluded from the analysis.

One possible approach after the detecting deviation from HWE in certain markers is to exclude the corresponding markers from the analysis. Our data show that excluding markers not in HWE from the analysis when non-differential genotyping errors are present does not have any effect on the type I error rate, which remains at the nominal significance level of 0.05, as for example in Figure 5 for a mean error rate of 15.6%. The observed inflation of the type I error rate due to differential genotyping errors is only moderately decreased when markers not in HWE are excluded from the analysis. Thus the type I error rate can be still around 0.2–0.3 for the Armitage trend test and 0.2–0.5 for the Mantel Statistic Using Haplotype Sharing in the presence of differential genotyping errors with a mean error rate per locus of 15.6% (Figure 5). This can also be observed for a mean error rate per locus of 8% (data not shown). As expected, the power of all three test statistics is decreased when markers not in HWE are excluded regardless of whether genotyping errors are differential or non-differential since the total number of SNPs studied is reduced (data not shown).

## Conclusion

We investigated the impact of genotyping errors on the performance of the Mantel Statistic Using Haplotype Sharing and the haplotype-based score test, haplo.score. Both are haplotype-based tests that have been applied to case-control data in population-based candidate gene association studies. The haplo.score is based on genotypes incorporated in a generalized linear model framework, but accounts for the uncertainty of haplotype phase within the calculations. On the other hand, the Mantel Statistic Using Haplotype Sharing needs the complete phase information of the individuals under study, i.e. the corresponding haplotype pair for each individual. For better comparison of the two haplotype-based methods we used the individuals' haplotype pairs determined via the EM-algorithm, implemented in R (haplo.em) [31]. This algorithm is also used by haplo.score and provides the same haplotype frequency distribution as incorporated in the haplo.score procedure. Haplo.score tests the global hypothesis of whether an association between any examined haplotype exists, whereas the Mantel Statistic Using Haplotype Sharing is a pointwise test that only incorporates the information of neighbouring markers. Hence, the comparison of these two methods might be hampered by the fact that they test different null hypotheses. We therefore additionally investigate the pointwise Armitage trend test as a third association test statistic. It has been shown that the trend test achieves greatest power, compared to other Chi-Squared tests, when there is no prior knowledge of the underlying disease model or in the presence of deviation from HWE [29]. Since the disease model in this simulation study is known to be recessive, the 2 × 2 (1 df) Chi-Squared test for independence with the count of the homozygote minor alleles (0, 0, 1) should be the most powerful test [34]. However, in the presence of genotyping errors, the advantage in power of this 1 df Chi-Squared-test over the Armitage trend test, for a known recessive disease model, could not be confirmed [29].

We find that in the presence of genotyping errors, with a mean error rate per locus of 0.2%, the type I error rate and the empirical power of all three statistics are not affected at all. The type I error rate is highly inflated only for high and differential genotyping error rates (8% and 15.6%). The magnitude of increase in type I error rate depends on the sample size, i.e. type I error rate is more inflated with a larger than with a smaller sample size, which was also previously reported by Moskvina et al. [25] and can be explained by the fact that differential errors are systematic errors ([35], p. 116). In the presence of high differential error rates, the two haplotype-based approaches were more sensitive, i.e. showed clearly higher type I error rates compared to the Armitage trend test.

Genotyping errors affect the number of haplotypes and shift the haplotype distribution towards an increased number of rare haplotypes. The amount of rare haplotypes increases with higher genotype error rates. The size of the study sample is also positively correlated with the number of additional haplotypes due to genotype errors. Our results indicate that this large amount of rare haplotypes is the reason for the inflation of the type I error rate of the Mantel Statistic Using Haplotype Sharing, since the statistic is based on all haplotypes, including the many rare ones. Our results agree with the previously reported observations of an inflated type I error rate in the presence of undetectable or sample-specific errors (differential errors) of former investigations [6-8,29].

The power gain for all three association test statistics for high and differential genotype errors is coherent in view of the above mentioned inflated type I error rate. We also observe a loss in power for nondifferential genotyping errors, as reported by Heid et al. [28]. On the other hand, the observation of Moskvina et al. [25] that the type I error rate of a haplotype-based association statistic is highly inflated even in the presence of a small genotyping error rate of less than 1% cannot be confirmed with this simulation. Moskvina et al. [25] draw this conclusion for markers in high LD and a relatively low minor allele frequency, whereas the markers we examined comprise haplotypes in two blocks of high LD and have MAFs of between 0.028 and 0.45. Nevertheless, we are able to confirm the effect of sample size on the type I error rate in the presence of genotyping errors, which Moskvina et al. [25] reported.

There has been a lively discussion on whether the exclusion from data analysis of markers that are not in HWE is an appropriate way to deal with genotyping errors [19-21]. Our results support the criticism of this approach, showing that the proportion of genotyping errors detected by testing for deviation from HWE can be quite low. Especially, in the case of common alleles, deviation of HWE is not a sufficient indicator for genotyping errors. We should point out that the chosen cut-off of p < 0.05 to indicate significant deviation from HWE is already very strict. Choosing a less stringent cut-off, as often suggested and conducted in practice, would further decrease the number of genotyping errors detected. Differential errors have been simulated to occur either only in cases or in different markers for cases and controls, as in most real situations. Thus, the test of deviation from HWE applied to controls only is not at all appropriate to detect such differential errors. Hence, the exclusion of markers not in HWE does not reduce the inflated type I error rate substantially. Furthermore, the exclusion of markers leads to a general loss in power, since markers truly associated with disease may also be eliminated.

We show that in the presence of a realistic amount of genotype errors (with a mean error rate per locus of 0.2%), all three examined methods to test association in candidate regions perform well. The Mantel Statistic Using Haplotype Sharing and the Armitage trend test hold their pointwise and the haplo.score its global nominal significance level of 5%. The power to detect the putative disease locus or a haplotype specific association remained high with 89%–94%.

## Authors' contributions

IMH and CL participated in the design of the incorporation of genotyping errors by means of misclassification models. VM and LB collaborated in the design and setting of the simulation scenarios. VM carried out the programming. JCC was involved planning the simulation study and revising the manuscript critically and gave final approval of the version to be published. All authors read and approved the final manuscript.

## References

[B1] Bross I (1954). Misclassification in 2 × 2 tables. Biometrics.

[B2] Saunders IW, Brohede J, Hannan GN (2007). Estimating genotyping error rates from Mendelian errors in SNP array genotypes and their impact on inference. Genomics.

[B3] Pompanon F, Bonin A, Bellemain E, Taberlet P (2005). Genotyping errors: causes, consequences and solutions. Nat Rev Genet.

[B4] Heid IM, Lamina C, Bongardt F, Fischer G, Klopp N, Huth C, Küchenhoff H, Kronenberg F, Wichmann HE, Illig T (2005). Wie gut können Haplotypen in den populationsbasierten KORA-Studien rekonstruiert werden?. Gesundheitswesen.

[B5] Liu W, Zhao W, Chase GA (2006). The impact of missing and erroneous genotypes on tagging SNP selection and power of subsequent association tests. Hum Hered.

[B6] Liu W, Yang T, Zhao W, Chase GA (2007). Accounting for genotyping errors in tagging SNP selection. Ann Hum Genet.

[B7] Quade SR, Elston RC, Goddard KA (2005). Estimating haplotype frequencies in pooled DNA samples when there is genotyping error. BMC Genet.

[B8] Zhu WS, Fung WK, Guo J (2007). Incorporating genotyping uncertainty in haplotype frequency estimation in pedigree studies. Hum Hered.

[B9] Govindarajulu US, Spiegelman D, Miller KL, Kraft P (2006). Quantifying bias due to allele misclassification in case-control studies of haplotypes. Genet Epidemiol.

[B10] Gordon D, Finch SJ, Nothnagel M, Ott J (2002). Power and sample size calculations for case-control genetic association tests when errors are present: application to single nucleotide polymorphisms. Hum Hered.

[B11] Kang SJ, Gordon D, Finch SJ (2004). What SNP Genotyping Errors Are Most Costly for Genetic Associatin Studies. Genet Epidemiol.

[B12] Mitchell AA, Cutler DJ, Chakravarti A (2003). Undetected genotyping errors cause apparent overtransmission of common alleles in the transmission/disequilibrium test. Am J Hum Genet.

[B13] Gordon D, Heath SC, Liu X, Ott J (2001). A transmission/disequilibrium test that allows for genotyping errors in the analysis of single-nucleotide polymorphism data. Am J Hum Genet.

[B14] Gordon D, Haynes C, Johnnidis C, Patel SB, Bowcock AM, Ott J (2004). A transmission disequilibrium test for general pedigrees that is robust to the presence of random genotyping errors and any number of untyped parents. Eur J Hum Genet.

[B15] Becker T, Valentonyte R, Croucher PJ, Strauch K, Schreiber S, Hampe J, Knapp M (2006). Identification of probable genotyping errors by consideration of haplotypes. Eur J Hum Genet.

[B16] Cheng KF, Chen JH (2007). A simple and robust TDT-type test against genotyping error with error rates varying across families. Hum Hered.

[B17] Gordon D, Haynes C, Yang Y, Kramer PL, Finch SJ (2007). Linear trend tests for case-control genetic association that incorporate random phenotype and genotype misclassification error. Genet Epidemiol.

[B18] Gordon D, Yang Y, Haynes C, Finch SJ, Mendell NR, Brown AM, Haroutunian V (2004). Increasing power for tests of genetic associatin in the presence of phenotype and/or genotype error by use of double-sampling. Stat Appl Genet Mol Biol.

[B19] Leal SM (2005). Detection of genotyping errors and pseudo-SNPs via deviations from Hardy-Weinberg equilibrium. Genet Epidemiol.

[B20] Cox DG, Kraft P (2006). Quantification of the power of Hardy-Weinberg equilibrium testing to detect genotyping error. Hum Hered.

[B21] Teo YY, Fry AE, Clark TG, Tai ES, Seielstad M (2007). On the usage of HWE for identifying genotyping errors. Ann Hum Genet.

[B22] Knapp M, Becker T (2004). Impact of genotyping errors on type I error rate of the haplotype-sharing transmission/disequilibrium test (HS-TDT). Am J Hum Genet.

[B23] Sha Q, Dong J, Jiang R, Chen HS, Zhang S (2005). Haplotype sharing transmission/disequilibrium tests that allow for genotyping errors. Genet Epidemiol.

[B24] Moskvina V, Schmidt KM (2006). Susceptibility of biallelic haplotype and genotype frequencies to genotyping error. Biometrics.

[B25] Moskvina V, Craddock N, Holmans P, Owen MJ, O'Donovan MC (2006). Effects of differential genotyping error rate on the type I error probability of case-control studies. Hum Hered.

[B26] Schaid DJ, Rowland CM, Tines DE, Jacobson RM, Poland GA (2002). Score tests for association between traits and haplotypes when linkage phase is ambiguous. Am J Hum Genet.

[B27] Beckmann L, Thomas DC, Fischer C, Chang-Claude J (2005). Haplotype sharing analysis using Mantel statistics. Hum Hered.

[B28] Heid IM, Lamina C, Küchenhoff H, Fischer G, Klopp G, Kolz M, Grallert H, Vollmert C, Wagner S, Huth C, Müller J, Müller M, Hunt SC, Peters A, Paulweber B, Wichmann HE, Kronenberg F, Illig F (2008). Estimating the single nucleotide polymorphism genotype misclassification from routine double measurements in a large epidemiologic sample. Am J Epi.

[B29] Ahn K, Haynes C, Kim W, Fleur RS, Gordon D, Finch SJ (2007). The effects of SNP genotyping errors on the power of the Cochran-Armitage linear trend test for case/control association studies. Ann Hum Genet.

[B30] Heid IM, Wagner SA, Gohlke H, Iglseder B, Mueller JC, Cip P, Ladurner G, Reiter R, Stadlmayr A, Mackevics V, Illig T, Kronenberg F, Paulweber B (2006). Genetic architecture of the APM1 gene and its influence on adiponectin plasma levels and parameters of the metabolic syndrome in 1,727 healthy Caucasians. Diabetes.

[B31] Sinnwell JP, Schaid DJ, Yu Z haplo.stats: Statistical Analysis of Haplotypes with Traits and Covariates when Linkage Phase is Ambiguous. R package version 131.

[B32] Stephens M, Smith NJ, Donnelly P (2001). A New Statistical Method for Haplotype Reconstruction from Population Data. Am J Hum Genet.

[B33] Ziegler A, König IR (2006). A Statistical Approach to Genetic Epidemiology. Weinheim: Wiley-VCH.

[B34] Slager SL, Schaid DJ (2001). Case-control studies of genetic markers: power and sample size approximations for Armitage's test for trend. Hum Hered.

[B35] Rothman KJ, Greenland S (1998). Modern Epidemiology.

